# Controlling the surface charge of simple viruses

**DOI:** 10.1371/journal.pone.0255820

**Published:** 2021-09-10

**Authors:** A. L. Duran-Meza, M. V. Villagrana-Escareño, J. Ruiz-García, C. M. Knobler, W. M. Gelbart

**Affiliations:** 1 Department of Chemistry and Biochemistry, University of California, Los Angeles, California, United States of America; 2 Laboratorio de Física Biológica, Instituto de Física, Universidad Autónoma de San Luis Potosí, San Luis Potosí, SLP, México; University of California, Riverside, UNITED STATES

## Abstract

The vast majority of plant viruses are unenveloped, *i*.*e*., they lack a lipid bilayer that is characteristic of most animal viruses. The interactions between plant viruses, and between viruses and surfaces, properties that are essential for understanding their infectivity and to their use as bionanomaterials, are largely controlled by their surface charge, which depends on pH and ionic strength. They may also depend on the charge of their contents, i.e., of their genes or–in the instance of virus-like particles–encapsidated cargo such as nucleic acid molecules, nanoparticles or drugs. In the case of enveloped viruses, the surface charge of the capsid is equally important for controlling its interaction with the lipid bilayer that it acquires and loses upon leaving and entering host cells. We have previously investigated the charge on the unenveloped plant virus Cowpea Chlorotic Mottle Virus (CCMV) by measurements of its electrophoretic mobility. Here we examine the electrophoretic properties of a structurally and genetically closely related bromovirus, Brome Mosaic Virus (BMV), of its capsid protein, and of its empty viral shells, as functions of pH and ionic strength, and compare them with those of CCMV. From measurements of both solution and gel electrophoretic mobilities (EMs) we find that the isoelectric point (pI) of BMV (5.2) is significantly higher than that of CCMV (3.7), that virion EMs are essentially the same as those of the corresponding empty capsids, and that the same is true for the pIs of the virions and of their cleaved protein subunits. We discuss these results in terms of current theories of charged colloidal particles and relate them to biological processes and the role of surface charge in the design of new classes of drug and gene delivery systems.

## Introduction

The charge on an unenveloped virus plays a significant role in its interaction with biological membranes. For example, driven by electrostatic interactions, picornaviruses such as Hepatitis A, which do not code for any membrane proteins, can become enveloped in host-derived lipid bilayers as they exit from cells [1]. Such wrapping is distinct from that in many enveloped viruses in which the lipid layer is supported by transmembrane glycoproteins that are bound to the viral capsid exterior. It has been shown as well that the use of adenoviruses in gene delivery can be enhanced by wrapping them in “artificial” lipid membranes [2]. The direct functionalization of a capsid has also been employed to provide specific interactions with a substrate, but even in this case the interactions can be affected by the underlying surface charge [3]. The formation of viral multilayers on solid surfaces can be controlled by pH-driven changes in charge [4] and the formation of multilayer shells by capsid proteins can be understood in terms of the interactions between bending elasticity and capsid surface charge [5]. Quite generally the utilization of viruses and virus-like particles (VLPs) in biotechnology often depends on their charge.

To have a quantitative understanding of the factors that control the charge on capsids and to employ this knowledge to enable them to generate enveloped VLPs, we have undertaken studies of the electrophoretic mobilities (EMs) of two similar bromoviruses, CCMV (Cowpea Chlorotic Mottle Virus) and BMV (Brome Mosaic Virus) [6]. They each have a tripartite genome consisting of three positive-sense RNAs that are packaged in separate unenveloped 26-nm diameter capsids made up of 180 identical proteins (T = 3 Caspar-Klug triangulation numbers). The viruses can be self-assembled in vitro when the purified capsid protein (CP) and viral RNAs are mixed under appropriate buffer conditions [7]. Moreover, spontaneous assembly of VLPs can also take place around heterologous RNAs [8], anionic polymers [9], negatively charged nanoparticles [10], nanolipospheres [11], and even negatively charged nanoemulsion droplets [12], making VLPs potentially useful vehicles for gene and drug delivery.

The CCMV and BMV CPs differ in length by only one amino-acid residue (CCMV 189, BMV 188) and have a 70% sequence homology. Both proteins have Arginine/Lysine-rich N-terminal arms–CCMV with 10 positive charges, and BMV with 9 –that extend into the capsid interior and interact electrostatically with negatively-charged cargo (RNA, in the native viruses). The basic residues in the N-terminal region have high pK_a_s and as a result remain positively charged over a wide range of pH. On the other hand, the charges of the acidic residues vary significantly with pH, leading to repulsive interactions between the CPs within the capsid shell at high pH; as the pH is lowered, the isoelectric point (pI) of the CPs is approached, allowing the attractive hydrophobic interactions between them to provide the “glue” that stabilizes the capsid [13].

The importance of electrostatic interactions in the assembly and stability of viruses and their pH dependence has been established by numerous experimental and theoretical studies [14–19]. In a recent investigation we explored the capsid charge of the CCMV virion by measurements of its EM, which show that its pI is 3.7 [14]. Much earlier, Bockstahler and Kaesberg [15] had used a Tiselius cell to determine the EM of BMV at an ionic strength of 0.10 M at 2°C at different pHs; they reported a pI of 7.9; ten years later, in contrast, Johnson, et al.^16^ found the BMV pI to be 4.8 using the same technique so the differences cannot be ascribed to the method of measurement. Despite the similarities of BMV and CCMV in gene composition and viral structure, there are significant differences in their electrostatic properties. In this study we examine the EM of BMV and its CP and N-terminal cleaved CP as a function of pH and ionic strength (I). Comparisons with CCMV allow us to understand how the CP structure controls the viral electrostatic properties. This information is needed not only to understand the physical basis of viral stability but also to determine the optimal solution conditions for ensuring efficient in vitro self-assembly and packaging of heterologous RNA into VLPs.

The differences in pI of CCMV and BMV capsids–in particular, differences in the sign and magnitude of their surface charge at various pHs–are also important for the design of their VLPs for gene delivery and provide a baseline for using VLPs as templates on which an artificial lipid bilayer envelope can be self-assembled. Singh, *et al*. [2], for example, have demonstrated how electrostatic interactions drive the wrapping of negatively-charged Adenovirus particles by a mixture of positively-charged lipid (1,2-dioleoyl-3-trimethylammonium-propane (DOTAP)) and cholesterol. On the other hand, it has been shown that cationic lipids show cytotoxicity in many different cells [20]. For this reason we are interested in elucidating the surface charge of BMV VLPs in order to determine the conditions of pH and ionic strength for which an anionic lipid can wrap them, forming an enveloped virus-like particle (EVLP) with improved biocompatibility and providing a new surface to which targeting ligands can be added to selectively enhance cellular uptake.

Amino acid modification of capsid protein provides an additional means for controlling the surface charge of VLPs [21], as does the use of polymer/nucleic acid complexes [3,22]. More explicitly, Chatterji *et al*. [21] have shown that it is possible to tune and control the surface electrostatic properties of the Cowpea Mosaic Virus (CPMV) capsid as a function of pH by engineering six contiguous histidine residues into various locations on the virus capsid. Further, Dosta *et al*. [22] have described a method to modify the function, specificity and toxicity of siRNA-containing nanoparticles using cationic and anionic oligopeptide-modified poly(β-amino ester) polymers to expand the biophysical properties of these particles, such as size and–especially–surface charge. Surface charge tunability is a powerful strategy for controlling electrostatic interactions that reduce toxicity and enhance cellular uptake.

In the present work we report systematic measurements of the EMs of RNA-containing BMV and CCMV VLPs, of their empty capsids, and of their constituent capsid protein subunits, as functions of pH and of ionic strength, in both gels and solution. Despite the significant sequence homology between their capsid proteins, we find a large difference in their isoelectric points and in the magnitudes of their surface charge over a wide range of pH, attributable to differences in their tertiary and quaternary structures and the pK_a_s of their external residues. Gel and Dynamic Light Scattering (Zetasizer) measurements of their solution and gel EMs show them to be independent of their RNA content, providing a basis for the optimization of VLP surface charge density for gene delivery and for wrapping in charged lipid bilayers for controlling immunogenicity and targeting.

## Materials and methods

### Virus growth and purification

BMV was purified following established protocols that are similar to those employed for CCMV [23]. Virus was extracted from barley plants, grown with 18/6h light/dark cycles. The plants were infected by mechanically inoculating primary leaves with virus 7 days after sprouting, using 20 μl of a 0.2 μg/μl solution of BMV. The symptomatic uninoculated leaves were harvested ten days after inoculation and were stored at -80°C until use. The virus was extracted from 200 g of frozen leaves that were ground in 400 ml of Virus Extraction Buffer (500 mM sodium acetate, 80 mM magnesium acetate pH 4.5). The resulting solution was filtered through cheese cloth, the flowthrough was then centrifuged, the pellet was discarded, and the supernatant was ultracentrifuged on a 10% sucrose cushion and then purified in a 10–40% sucrose gradient. The final solution was centrifuged to separate the pellet containing the virus from the sucrose, and the concentration and purity were measured by UV-Vis Spectrophotometry (NanoDrop 2000C, Thermo Scientific, MA, USA). The virus was resuspended in Virus Suspension Buffer (VSB) (50 mM sodium acetate, 8 mM magnesium acetate, pH 4.5) and aliquoted at 1 mg/ml for storage at -80°C.

### Capsid protein purification

To purify the capsid protein we use the protocol described by Lavelle *et al*. [24] The purified virions were disassembled by dialysis in a Disassembly Buffer (500 mM CaCl_2_, 50 mM Tris-HCl pH 7.5, 1 mM EDTA pH 8.0, 1 mM DTT, 0.5 mM PMSF pH 7.5) for 12 h at 4°C with constant stirring. The solution was then ultracentrifuged (L-80 Beckman, CA, USA) at 543,000 g using a TLA-110 rotor for 126 min at 4°C. 300 μl aliquots were immediately taken, and their concentration was measured by UV-Vis spectrophotometry. Aliquots with a purity indicated by a protein:RNA ratio greater than 1.6 were chosen for this study. The protein was then dialyzed for 12 h against Protein Buffer (1M NaCl, 20 mM Tris-HCl pH 7.2, 1mM EDTA pH 8.0, 1 mM DTT, 1mM PMSF), stored at 4°C and used within 2 weeks.

### Empty capsid assembly

BMV empty capsids were assembled according to the procedure described by Pfeiffer and Hirth [25]. 25 μg of purified CP at a concentration of 0.5 μg/μl was dialyzed overnight against acetate buffer 0.3 M, pH 4.6 at 4°C.

### Electrophoretic gel measurements

1% Agarose gels were performed in an EasyCast™ B1A gel electrophoresis system (Thermo Scientific), using as electrophoresis buffer: 0.1M citrate buffer for pH 4 and 5, VSB for pH 4.5, and 0.1M phosphate buffer for pH 6 and above

### Electrophoretic mobility measurements

The EM measurements were performed with a Zetasizer NanoZS (Malvern, U.K.) at 25 ^o^C, using 800 μL aliquots of a 0.4 mg/mL solution. The virus was dialyzed in a 12 kDa dialysis membrane for 12 h in the desired pH buffer with stirring at 4°C and kept at that temperature until use. Each data point is the mean of 10 measurements, each of which was itself the mean of 10 measurements. The size distribution of each sample was measured, using the instrument in light scattering mode, before the mobility measurements.

### Preparation of CP lacking the N-terminus

The CP was cleaved by treatment with chymotrypsin using the procedure described by Vega-Acosta, *et al*. [14] The loss of the N terminus was verified by gel electrophoresis and MALDI TOF mass spectrometry, as shown in the S1 Fig.

## Results and discussion

### pH of electrophoresis buffer determines the gel electrophoretic mobility of CCMV and BMV virions

**Fig 1:**
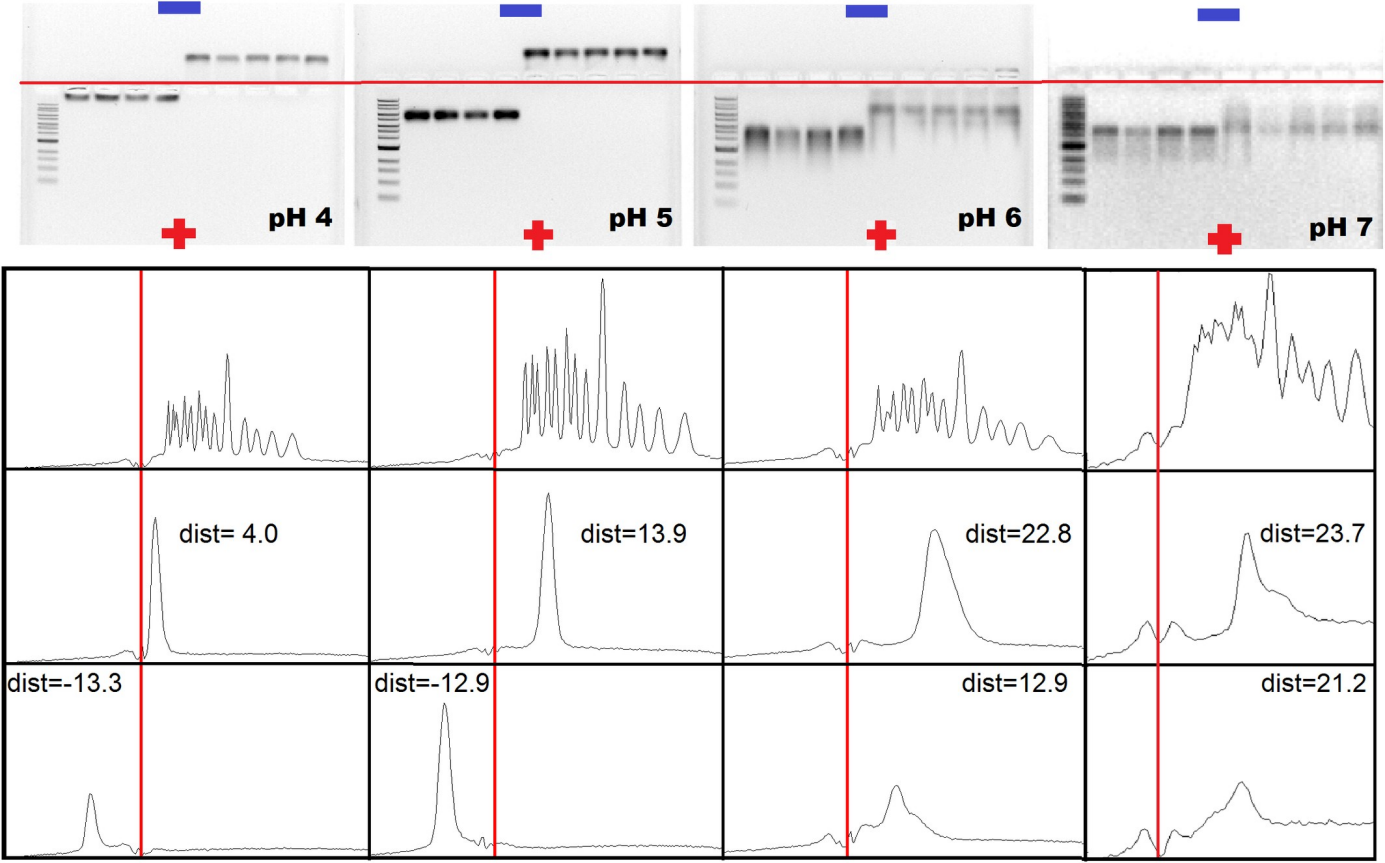
Top. Electrophoretic mobility patterns of CCMV and BMV in 1% agarose gels at pHs 4, 5, 6, and 7 as electrophoresis buffer. In each of the four gels, the samples were loaded as follows: 1-kb-extended DNA ladder (lane 1); CCMV in VSB (pH 4.5), CCMV dialyzed into pH 4, into pH 5, and into pH 6 (lanes 2, 3, 4 and 5, respectively); BMV in VSB (pH 4.5), BMV dialyzed into pH 4, 5, 6 and 7 (lanes 6, 7, 8, 9 and 10, respectively). The red horizontal lines specify the position of the wells. Bottom. Density plots of lanes 1, 2 and 6 to illustrate quantification of the distance that the virus has traveled in each case. The red vertical lines denote the positions of the well.

**Fig 2:**
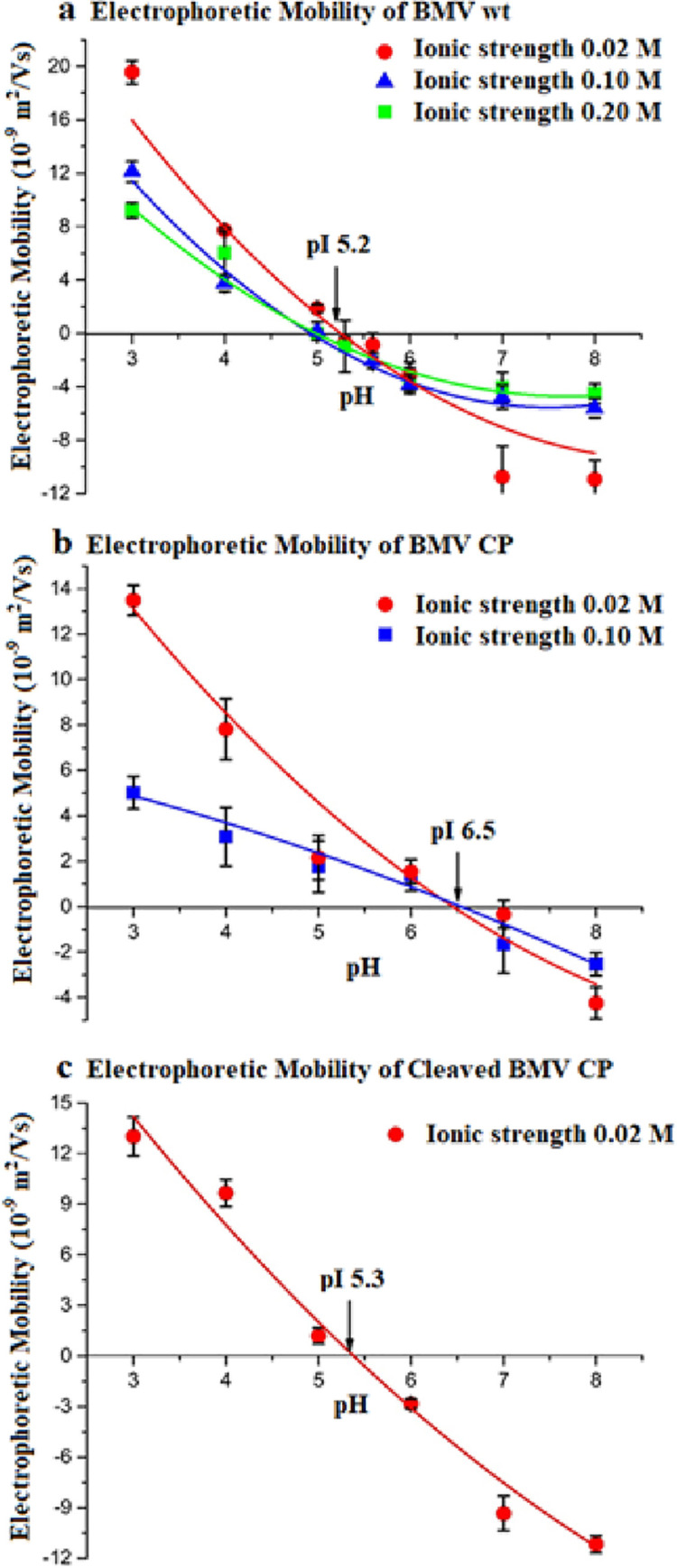
Electrophoretic mobility as a function of pH and ionic strength, for: (a) BMV; (b) BMV CP; and (c) Cleaved BMV CP. The lines, the results of a 2^nd^ order polynomial fit, are a guide to the eye.

Agarose gels, in which CCMV virions are compared alongside BMV virions in electrophoresis buffers at different pHs, point up dramatically the significant differences between the EMs of the viruses. The buffers for electrophoresis in the four gels shown in Fig 1 correspond to pHs of 4, 5, 6 and 7. In each gel the leftmost lane is a 1kb DNA ladder, and lanes 2–5 contain CCMV virions that have been dialyzed in the appropriate-pH buffer (see Methods) at pH 4.8, 5, 6 and 7, respectively, before being loaded into the gel. The fact that the virus migrates the same distance irrespective of the pH into which it was dialyzed demonstrates that its size and charge–and therefore its mobility–is determined by the pH of the electrophoresis buffer and not on its previous history. Similarly, although the BMV in lanes 6–10 of each of the four gels had first been dialyzed into buffers with pHs from pH 4 to 7, the band position depends only on the pH of the electrophoresis buffer. Note that in every case the CCMV bands lie below the well, i.e., they have moved toward the positive electrode because of their negative charge. The short distance travelled from the well at pH 4 is indicative of the close proximity to the isoelectric point for the virus at this pH. In contrast, at pH 4 and 5, the BMV moved in the opposite direction, toward the negative electrode, while at 6 and 7, like CCMV, it moved toward the positive electrode. Clearly, the pI for CCMV must lie lower than 4, and that for BMV between 5 and 6, consistent with what we report below (see Fig 2A) from direct measurements of the solution EMs.

The gels in Fig 1 differ from those shown in the Supporting Information in Vega-Acosta, *et al*. [14] who report that BMV has a positive surface charge at pH 5.5 and 7.0, whereas we find that it is negative in both cases. The difference is likely attributable to the electrophoresis buffer not being changed frequently enough in the earlier measurements.

### Solution measurements of the mobility of BMV virions, BMV CP and cleaved BMV CP

The gel results in Fig 1 are in accord with solution measurements of the EM with a Zetasizer. The instrument measures the Doppler frequency shift in light scattered from charged particles in solution whose velocity is imposed by the application of an electric field, which provide a detailed and precise picture of the EM and its dependence on pH and ionic strength. As shown in Fig 2A, and in accord with our gel studies, the pI of BMV is 5.2 and is essentially independent of the ionic strength. On either side of the pI, at higher and lower pH, the magnitude of the charge on the virus decreases with increasing ionic strength, a result of counterion condensation.

**Fig 3:**
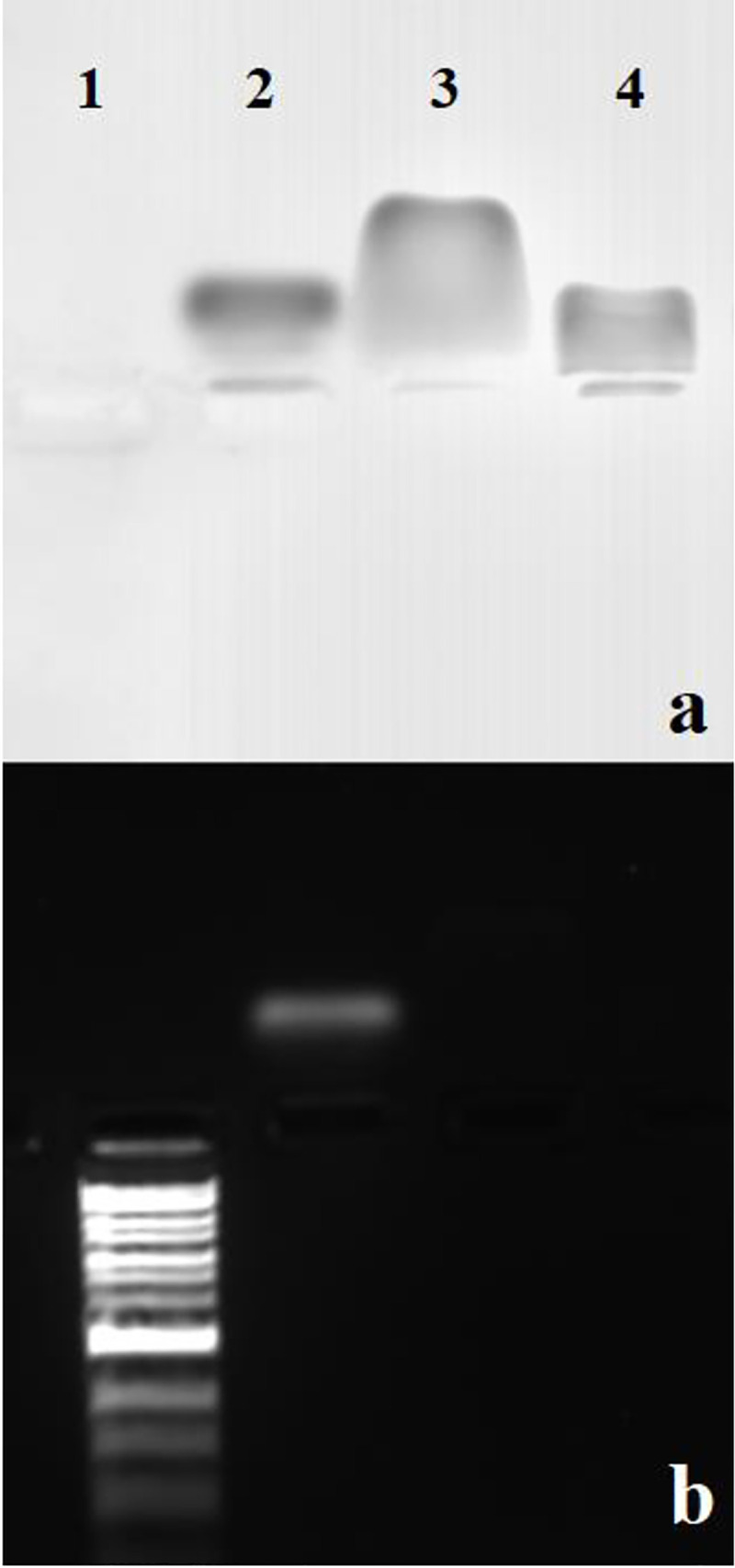
Comparison of wt BMV and empty BMV capsids by gel electrophoresis. The electrolysis was carried out at 4 ^o^C with a 0.3 M acetate electrophoresis buffer in a 0.8% agarose gel. As a control, the gel was stained with the nucleic-acid stain GelRed (b), followed by the protein stain Coomassie Blue (a). Lane 1, DNA1 kb ladder; lane 2, wt BMV; lane 3, BMV CP; and lane 4, BMV empty capsids.

The EM for the CP is shown in Fig 2B: its pI, 6.5, is significantly (1.3 units) higher than that of the virus (5.2), as is the case for CCMV CP and virus (4.8 versus 3.7). In the case of the cleaved BMV CP (Fig 2C), however, the pI is seen to be 5.2, essentially the same as that of the virus, consistent with the fact that charge on the capsid interior does not contribute to its EM. For CCMV, the virus and cleaved protein pIs are also close to one another (3.7 and 4.1). Similarly, we find that the gel EMs of wt and empty BMV capsids are the same: see Fig 3. Johnson, *et al*. [13] had also found that the solution EMs of CCMV and BMV virions are controlled by their surface charge and more recently, Chakravarty, *et al*. [26] have shown that BMV virions engineered to contain either BMV RNA 1 (3200 nt), 2 (2800 nt) or 3+4 (2900 nt) have indistinguishable gel EMs at pH 4 and are negatively charged.

### Electrophoretic mobility comparison between BMV and CCMV virus, wt CP and cleaved CP

**Fig 4:**
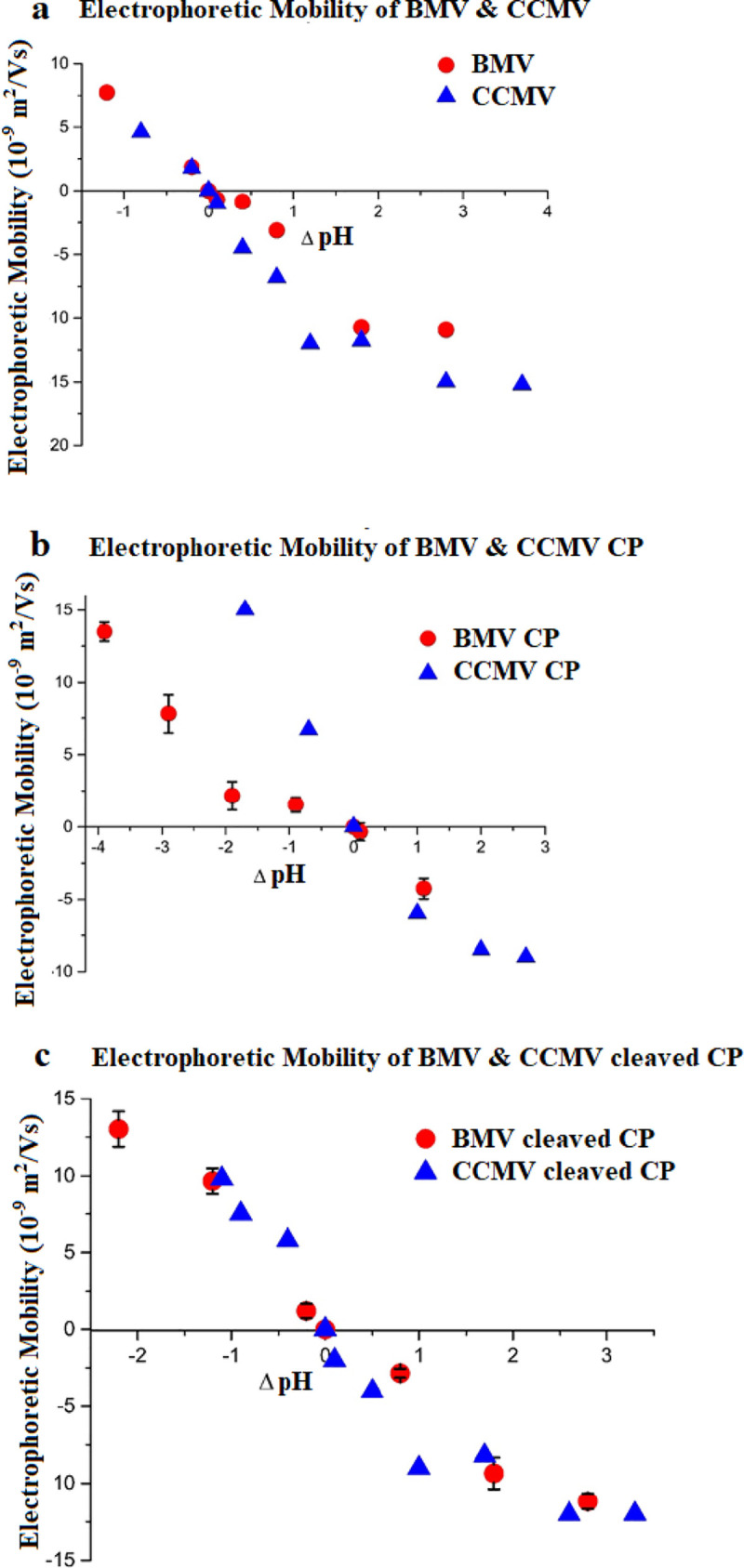
Electrophoretic mobility of the viruses, CPs and cleaved CPs at ionic strengths of 0.02 M, with the ordinate in each case shifted by the appropriate pI, i.e., ΔpH = pH–pI, as a function of pH and ionic strength, for: (a) BMV and CCMV; (b) BMV and CCMV CP; and (c) Cleaved BMV and CCMV CP.

To highlight the differences between BMV and CCMV, we have plotted in Fig 4A–4C the EMs of the viruses, CPs and cleaved CPs, at ionic strengths of 0.02 M, with the ordinate in each case shifted by the appropriate pI, i.e., ΔpH = pH—pI. With the exception of the wt CP, Fig 4B–where the higher charge on the N terminus of CCMV, even though it is only one charge, leads to a significantly higher positive mobility below the pI–the behaviors of the viruses and their cleaved CPs are not significantly different *when scaled in this way*. Note, however, that the very different charges on the BMV and CCMV capsids *at any particular pH* are expected to have biological consequences in the cellular context where significant pH variation throughout the range 4.5 to 8 is not uncommon in a wide variety of mammalian cells [27]. For example, in studies of breast cancer cells Nuñez-Rivera et al. [28]. observed a slightly higher efficiency of internalization of BMV compared to CCMV, each containing siRNA for therapeutic purposes. More strikingly, the CCMV particles showed a high degree of activation of macrophages whereas BMV produced virtually no immunologic response, with the difference attributed to the charge difference between the particles.

The relationship between the EM, a dynamic quantity, and the charge distribution on a virus or virus-like particle is complicated because it depends not only on the structure and positions of the charged residues but also on the accessibility of the residues to the solvent. This has been addressed by Ohshima and collaborators [29] who defined a “flow penetration depth” λ which when small correlates the EM with the surface charge density and is insensitive to the charges within the particle interior. The connection between λ and structure, however, is not obvious; for example, recent studies of the bacteriophage MS2 [30] show that its EM is sensitive to the interior charges. A similar result was found for phage phi29 in AFM studies by Hernando-Perez, *et al*. [31], but in contrast they found for adenovirus that its DNA did not affect the overall charge. As clearly shown by our experiments and those of Johnson, *et al*. [16], for CCMV *and* BMV, the EM is insensitive to the packaged RNA. It is thus useful to consider them as models that link EM and surface charge.

As discussed by Vega-Acosta, *et al*. [14] it is difficult to connect quantitatively the EM of the virion to the number of charges on its outside. They describe how an estimate of the charge can be obtained by utilizing the Henry equation [32], which is appropriate for a spherical colloid. According to this model, the EM is proportional in first order to the ratio of the charge to the diameter. Thus, since CCMV and BMV have identical diameters, the BMV charge can be calculated from the ratio of their EMs. Vega-Acosta, et al. [14] reported that at an ionic strength of 0.02 M, estimates of the charge on CCMV made with the Henry equation range from -100 at pH 5 to -160 at pH 7. Taking the ratios of the EMs we find a charge of +20 for BMV at pH 5 and– 100 at pH 7.

In the absence of EM measurements, structure-based estimates of capsid charge have been employed. The VIPER data base [6] includes values of the viral surface charge determined by summing the charges of the surface-exposed residues without consideration of pH or ionic strength, yielding -120 charges for CCMV and +120 for BMV. More detailed estimation procedures have been described by Lošdorfer Božič, *et al*. [33], who use a double-shell model and the VIPER database to calculate the radial mass distribution of the capsid and determine inner and outer radii, defined as inner and outer half maxima of the mass distribution. They then identify the surface charged residues–those that lie at radii in excess of the outer radius and determine the charge of each at a specified pH using the Henderson-Hasselbach equation. At pH 7.4 they obtain total surface charge values of -360 for CCMV and -60 for BMV and pIs of 4 and 6, respectively, qualitatively consistent with our observations.

The results reported here have immediate practical consequences relevant to the use of VLPs as gene delivery vectors. In particular, we have shown that therapeutic-RNA-containing VLPs can be reconstituted in vitro by mixing CCMV or BMV CP with the RNA of interest as long as its length is between 2500 and 4000nt.^8^ Even though the CP is from a plant virus, its mRNA content is made available to the ribosomal machinery of mammalian cells, with subsequent expression of either a reporter (e.g., EYFP or luciferase) or therapeutic (e.g., viral or cancer antigen) protein [34]. Building on the results of the present work we are now in a position to modify the surface charge of the VLPs in several ways, to further facilitate their in vivo efficacy as gene delivery systems.

First, we can exploit the relatively high pI (5.2) of BMV to prepare (at pH of 5, say) positively-charged VLPs containing therapeutic RNA, thereby enabling the wrapping of these VLPs with lipid bilayers consisting predominantly of neutral lipids except for a “pinch” of anionic lipids to electrostatically stabilize the enveloped virus-like particle. In this way we avoid the cytotoxicity associated with the cationic lipid that would have been necessary for wrapping CCMV at reasonable pH. The lipid bilayer envelope can then be functionalized with ligands and fusogens for enhancing the binding and uptake of the particles by targeted mammalian cells.

Alternatively, we can forego the wrapping of the VLPs by oppositely-charged lipid bilayers and instead conjugate the VLP CPs directly to membrane-binding moieties such as the amphoteric RGD (arginine-glycine-aspartic acid) motif or to positively-charged arginine/lysine-rich cell-penetrating-peptide sequences. The role of positive surface charge in enhancing cellular uptake of VLPs can be exploited even more simply by single-point mutations in the exterior loops of BMV proteins, e.g., by replacing the neutral residues at positions 63 or 163 by an arginine or lysine and assembling capsids from stoichiometric mixes of wildtype and mutant proteins to give the desired positive surface charge density, even at neutral pHs where it would otherwise be negative.

Finally, returning to the EVLP strategy, some of the anionic lipid could be replaced by amphiphilic RGD molecules^34^ consisting of long hydrocarbon “tails” with RGD “heads”. In all of these instances the biological demands on the VLPs are being satisfied by control of the surface charge distributions.

## Conclusions

The surface charge on a virus or virus-like particle–along of course with its amino acid modification and conjugation to ligands–is the most important determinant of its solubility and of its interactions with cell membranes. Measuring both gel and solution EMs we have found consistent agreement between the two approaches. Each shows the same significant differences between the pH-dependent charges of the outer surfaces of the capsids of two closely-related viruses–CCMV and BMV–both of which have the same 180-subunit icosahedrally-symmetric structures of the same size^6^. The differences in surface charge appear most dramatically in our measurement of a much lower isoelectric point for CCMV (3.7) than for BMV (5.2). Despite the significant differences in pI values, plots of EM versus pH for the two viruses lie on top of one another when plotted versus Δ*pH*≡*pH−pI*, and for both CCMV and BMV the EMs of their capsids are essentially independent of whether they are empty or contain viral RNA. Further, for both CCMV and BMV the pIs of the virus particles are essentially the same as those of the capsid protein subunits whose cationic N-termini have been cleaved, again pointing up the lack of dependence of capsid EM on internal charge (of protein or RNA). Finally, these experimental results can be understood in terms of the pK_a_s of the exterior charged residues and by the Henry equation relating EMs to the exterior surface charges of colloidal particles.

## Supporting information

S1 Fig a) 12% SDS Polyacrylamide gel electrophoresis (SDS-PAGE). Lane 1: Protein Ladder, lanes 2 and 3: BMV CP wild type, lanes 4 and 5 N-terminus cleaved BMV CP and lanes 6 and 7 BMV CP wild type, (the gel was stained with Coomassie Blue). Mass spectrum of b) BMV CP wild type and c) N-terminus cleaved BMV.
(DOCX)

S1 Raw images (PDF)
