# Supplementary material for: Controlling the surface charge of simple viruses
Source: PLoS One. 2021 Sep 10;16(9):e0255820. doi: 10.1371/journal.pone.0255820 (PMC8432797; doi:10.1371/journal.pone.0255820)
Supplement: S1 Fig — a) 12% SDS Polyacrylamide gel electrophoresis (SDS-PAGE). Lane 1: Protein Ladder, lanes 2 and 3: BMV CP wild type, lanes 4 and 5 N-terminus cleaved BMV CP and lanes 6 and 7 BMV CP wild type, (the gel was stained with Coomassie Blue). Mass spectrum of b) BMV CP wild type and c) N-terminus cleaved BMV. (DOCX) [file pone.0255820.s001.docx]

Supplementary Material


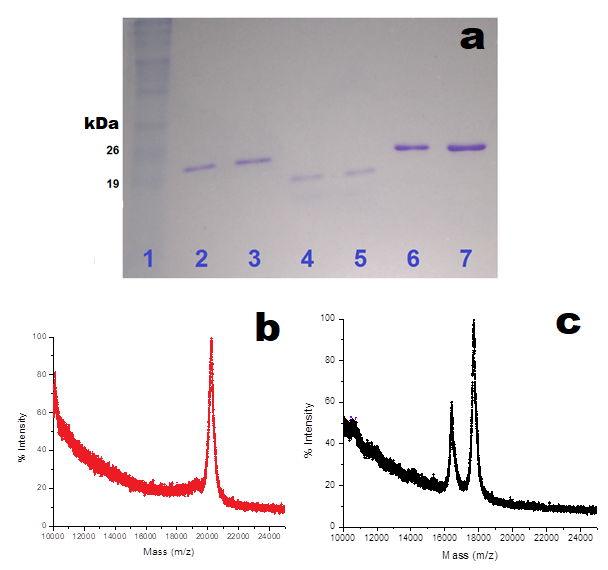


Fig. S1. a) 12% SDS Polyacrylamide gel electrophoresis (SDS-PAGE). Lane 1: Protein Ladder, lanes 2 and 3: BMV CP wild type, lanes 4 and 5 N-terminus cleaved BMV CP and lanes 6 and 7 BMV CP wild type, (the gel was stained with Coomassie Blue). Mass spectrum of b) BMV CP wild type and c) N-terminus cleaved BMV.
