# Supplementary material for: Controlling the surface charge of simple viruses
Source: PLoS One. 2021 Sep 10;16(9):e0255820. doi: 10.1371/journal.pone.0255820 (PMC8432797; doi:10.1371/journal.pone.0255820)
Supplement: S1 Raw images — (PDF) [file pone.0255820.s002.pdf]

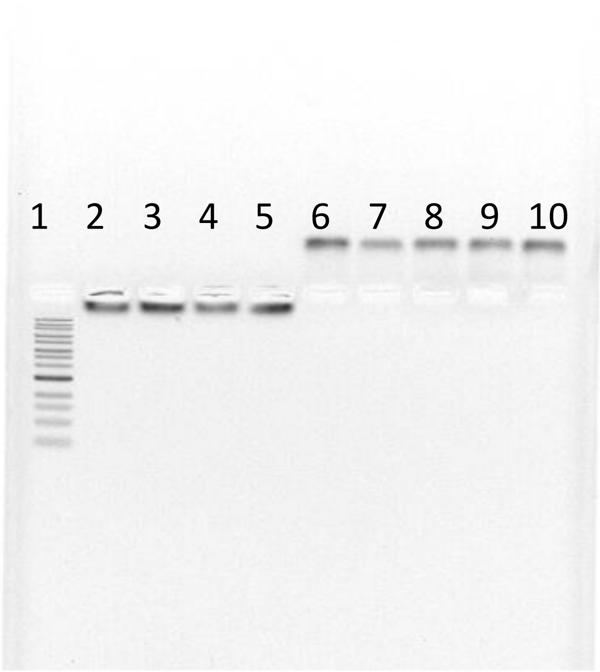

From Figure 1. 1% agarose gels at pHs 4. 1-kb-extended DNA ladder (lane 1); CCMV in VSB (pH 4.5), CCMV dialyzed into pH 4, into pH 5, and into pH 6 (lanes 2, 3, 4 and 5, respectively); BMV in VSB (pH 4.5), BMV dialyzed into pH 4, 5, 6 and 7 (lanes 6, 7, 8, 9 and 10, respectively). The gels were stained with gelred and Imaged in a gel imager azure C300 (Dublin, Ca, USA)

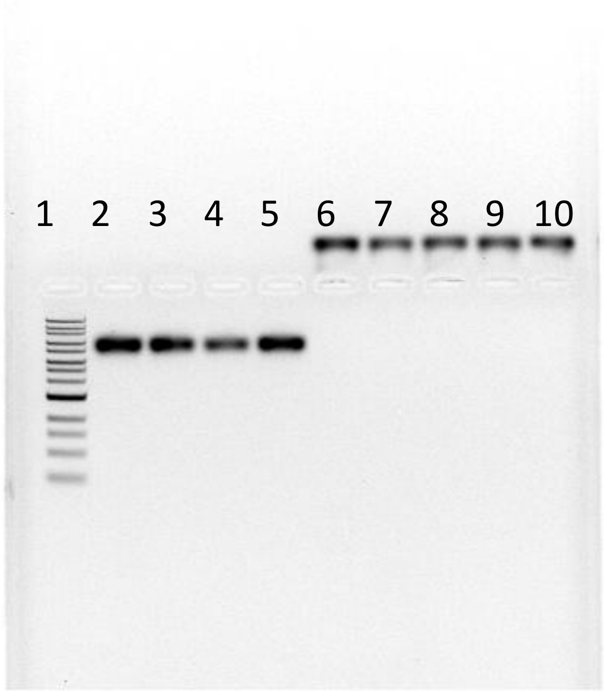

From Figure 1. 1% agarose gels at pHs 5. 1-kb-extended DNA ladder (lane 1); CCMV in VSB (pH 4.5), CCMV dialyzed into pH 4, into pH 5, and into pH 6 (lanes 2, 3, 4 and 5, respectively); BMV in VSB (pH 4.5), BMV dialyzed into pH 4, 5, 6 and 7 (lanes 6, 7, 8, 9 and 10, respectively). The gels were stained with gelred and Imaged in a gel imager azure C300 (Dublin, Ca, USA)

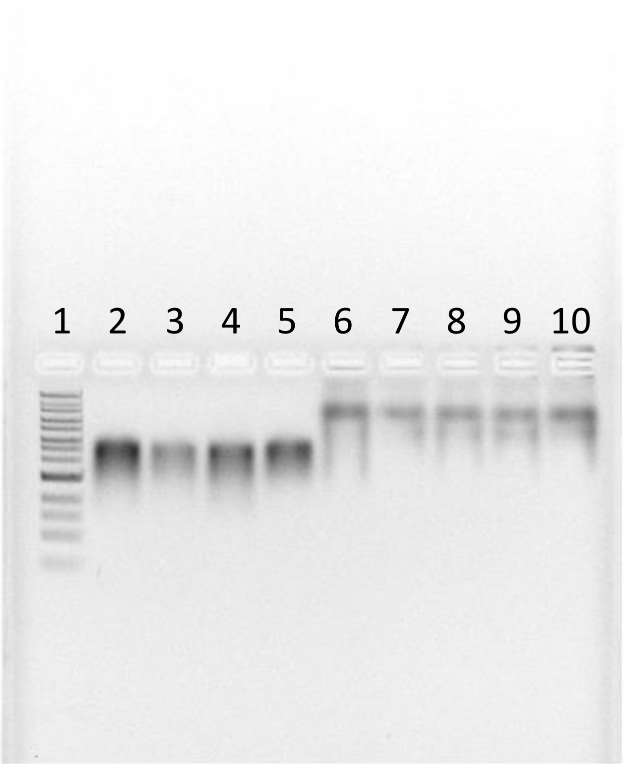

From Figure 1. 1% agarose gels at pHs 6. 1-kb-extended DNA ladder (lane 1); CCMV in VSB (pH 4.5), CCMV dialyzed into pH 4, into pH 5, and into pH 6 (lanes 2, 3, 4 and 5, respectively); BMV in VSB (pH 4.5), BMV dialyzed into pH 4, 5, 6 and 7 (lanes 6, 7, 8, 9 and 10, respectively). The gels were stained with gelred and Imaged in a gel imager azure C300 (Dublin, Ca, USA)

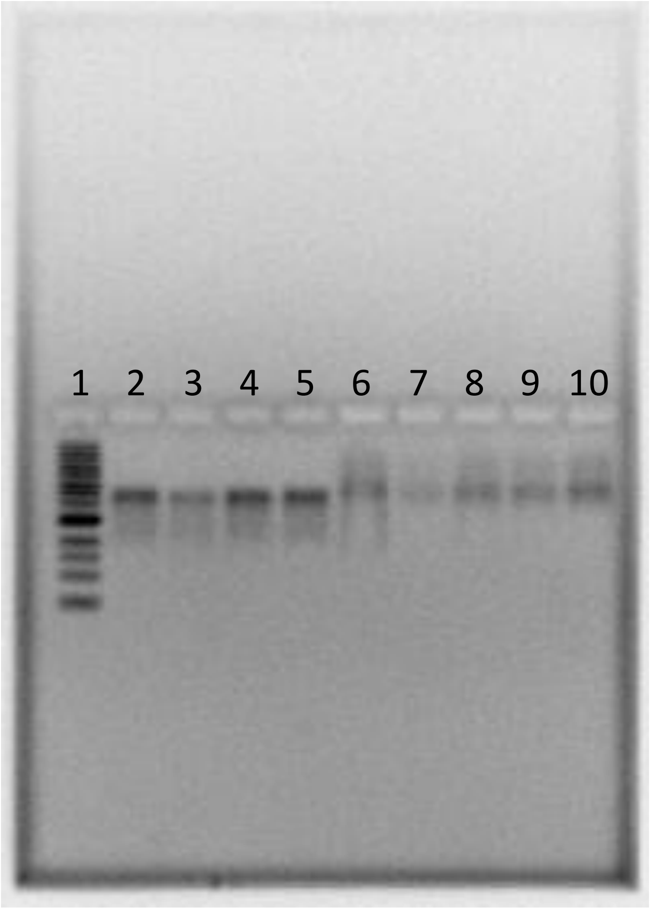

From Figure 1. 1% agarose gels at pHs 7. 1-kb-extended DNA ladder (lane 1); CCMV in VSB (pH 4.5), CCMV dialyzed into pH 4, into pH 5, and into pH 6 (lanes 2, 3, 4 and 5, respectively); BMV in VSB (pH 4.5), BMV dialyzed into pH 4, 5, 6 and 7 (lanes 6, 7, 8, 9 and 10, respectively). The gels were stained with gelred and Imaged in a gel imager azure C300 (Dublin, Ca, USA)

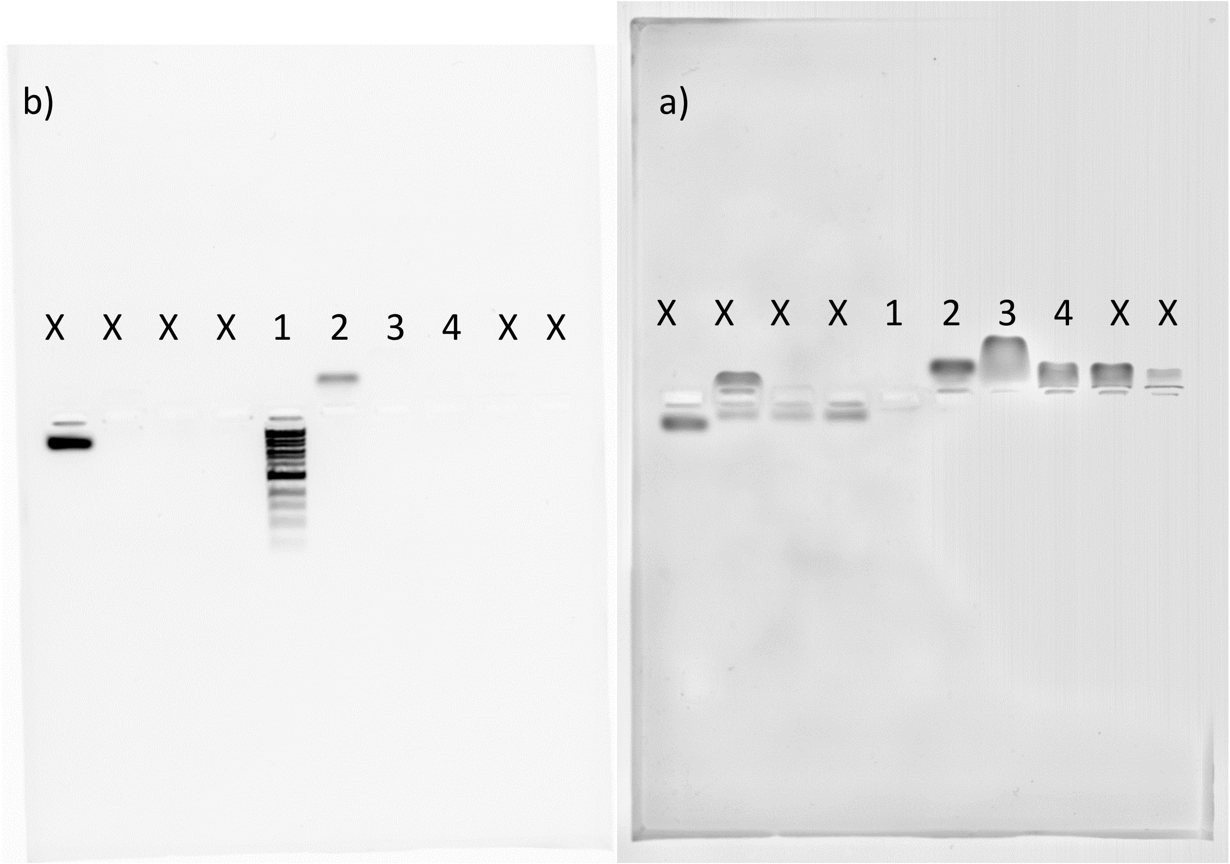

From Figure 3. 0.8 Agarose gel carried out at 4 °C with a 0.3 M acetate, the gel was stained with the nucleic-acid stain GelRed (b), followed by the protein stain Coomassie Blue (a). Lane 1, DNA1 kb ladder; lane 2, wt BMV; lane 3, BMV CP; and lane 4, BMV empty capsids. The gels were Imaged in a gel imager azure C300 (Dublin, Ca, USA)

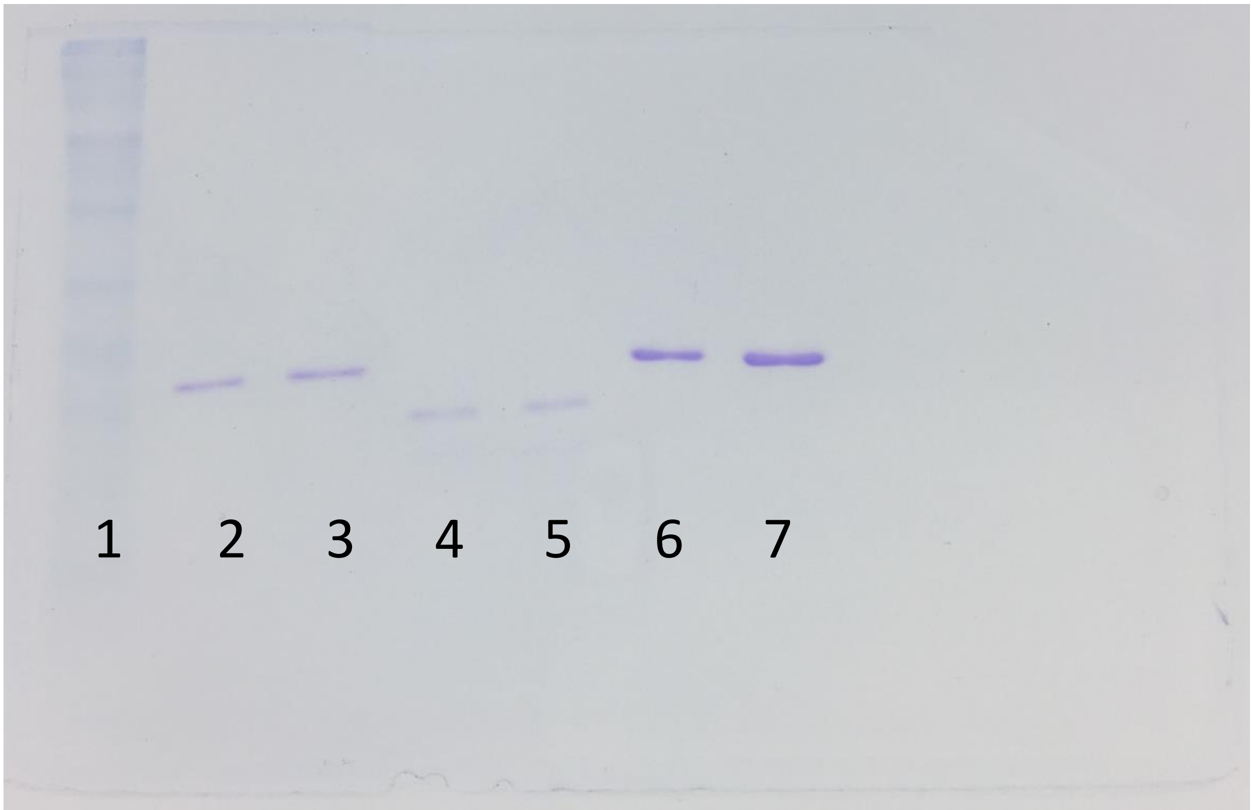

From Fig. S1. 12% SDS Polyacrylamide gel electrophoresis (SDS-PAGE). Lane 1: Protein Ladder, lanes 2 and 3: BMV CP wild type, lanes 4 and 5 N-terminus cleaved BMV CP and lanes 6 and 7 BMV CP wild type, (the gel was stained with Coomassie Blue). Imaged with a digital camara
